# Inflammation

**Published:** 1872-05

**Authors:** 


### EDITORIAL.

### Inflammation.

The etyomology of this term in some measure reveals the concept of the process, thus nominated by those who first designated it as inflammation. *In* and *flammo*, two Latin words, signifying an internal burning, or burning within.

So far from finding fault with early observers, we should be thankful to them for the brilliant conceptions of this process that led them to adopt the term as significative of the process.

Even to this day, there is little agreement among investigators or teachers, of the character of the process itself, the best methods of expressing it, describing it, and explaining it so that the mind may clearly conceive it, and perspicuously possess it.

This process, being a perversion of normal nutrient activity, involves all the factors of function, so that, if we would comprehend the aberrant functioning, we must possess a knowledge of the bodies and processes that elaborate function.

The time was not long since that the highest conception of function was "the work of organ, perceptible to unaided sight," such as a gland, a muscle, or an apparatus; respiratory, circulatory, or digestive.

But, since the discovery of the microscope and the manufacture of good objectives, the elements of these apparatuses and organs upon which they depend for the power to elaborate the function proper to them have been revealed and denominated cells.

Every body and process is the confluence of force and form, and each individual observer is the result of an equation or conjunction of consciousness and physics. It requires some preparation and study to master these propositions just named, that lay as ground or basis of body and being, simple or multiple.

Those who discovered, and first described cells, incline too much to physical and mechanical aspects, and hence endowed them with external skin or wall, fluid contents, and sometimes nucleus and nucleolus, in accordance with the facts before them. The later observations inform us of the soft-solid character of cells, without physical deferentiation of cell wall and parenchyma, and suggest endosmosis and exosmosis as modes of the process of nutrition of these elements, through which all the tissues are produced and multiplied in the interest of the organs that together constitute the system.

This endosmosis and exosmosis involve more than percolation, or a simple passing in of pabulum, and out of debris.

The old concept of feeding pabulum to cell partook too largely of a difference between cell wall and contents (as appropriators of pabulum and rejectors of debris) and pabulum itself. It is probable that the quantities of pabulum, received by the cell as a whole in the act of nutrition, is the conjoint act of the two, of placing pabulum, and displacing debris, throughout the entire functioning portion of the cell.

The maximum amount of change taking place at the center or nucleus. This is evinced in the feeding of colored pabulum to cells, which first affects the nucleus, and also in anthracosis when the inert particles of coal, incapable of dissolving or absorbing, take up their station in the formed material of connective tissue of alveolar septa of living and other alveoli; the latter exemplified in the tatooing process; in which these essentially foreign particles remain without exciting or influencing nutrition in any manner.

The appropriation of pabulum and the rejection of debris are the essential constituents of cell nutrition, and so stimulate the respiration of the body as a whole that they may be instanced as mutually explanatory.

Conjunctivitis, periostitis, parperitonitis and all the names of organs and tissues ending in *itis* throughout pathological literature are intended to convey to the mind of the reader the condition of perversion of nutrient action of these organs and tissues that has been and continues to be denominated inflammation.

If we regard the fluid and corpuscular blood as tissue, then hematitis is as legitimate nomenclature as ostitis, ophthalmitis and the best of the itises; but if blood, fluid and corpuscular, be regarded as pabulum only, out of which tissues are formed and fed then hematitis or inflammation of the blood would only lead us astray.

Inflammation confessedly is a local disturbance of functional activity, as all these terms just referred to signify.

The territory, or so to speak, depth of inflammatory activity, has a limit in accordance with the tissues involved, and progresses by continuity of kind of tissues in preference to contiguity of diseased tissues.

When it does spread by contiguity, it depends upon arrest of nutrition in the adjoining structure by pressure or mechanical invasion, and not by the specific cause of the inflammatory act. When the juices of the flesh are in a perfectly healthy condition, wounds heal without the intervention of the inflamatory process; but this process becomes a necessary step of healing in case of deterioration to a considerable degree of the inter and intra-cellular fluids. A.

